# Thermodynamic and Kinetic Analysis of Molecular Conformational Dynamics in a Riemannian Framework

**DOI:** 10.1021/acs.jpca.5c05362

**Published:** 2026-01-26

**Authors:** Ashkan Fakharzadeh, Curtis Goolsby, Emad Tajkhorshid, Mahmoud Moradi

**Affiliations:** † Theoretical and Computational Biophysics Group, NIH Resource for Macromolecular Modeling and Visualization, Beckman Institute for Advanced Science and Technology, 416418 University of Illinois Urbana−Champaign, Urbana, Illinois 61801, United States; ‡ Department of Biochemistry, and Center for Biophysics and Quantitative Biology, University of Illinois Urbana−Champaign, Urbana, Illinois 61801, United States; § Department of Chemistry and Biochemistry, 3341 University of Arkansas, Fayetteville, Arkansas 72701, United States; ∥ Department of Chemistry, University of Illinois Urbana−Champaign, Urbana, Illinois 61801, United States

## Abstract

We have formulated a Riemannian framework for describing the geometry of collective variable (CV) spaces in molecular simulations and demonstrate its applicability through both toy model potentials and a biomolecular example. This formalism addresses significant theoretical challenges arising from the inherent nonlinearity of CV transformations, ensuring critical quantities such as the potential of mean force (PMF), minimum free energy path (MFEP), and rate constant remain invariant under coordinate transformations. Our framework identifies and addresses the noninvariance issues of conventional PMF definitions, clearly illustrating their limitations through simple illustrative examples. To overcome these issues, we introduce invariant definitions of PMF and MFEP using Riemannian geometry. Moreover, we propose a generalized Riemannian diffusion model applicable to diffusive dynamics within CV spaces, allowing rigorous estimation of kinetic properties. Through this model, we derive practical numerical methods for determining the PMF, diffusion constant, metric tensor, and transition rates from unbiased simulations conducted along identified transition pathways. By integrating Bayesian approaches with the Riemannian framework, our method provides a statistically robust technique for accurately calculating free energy landscapes and transition kinetics, thereby enhancing the reliability and interpretability of biomolecular simulations.

## Graphical abstract

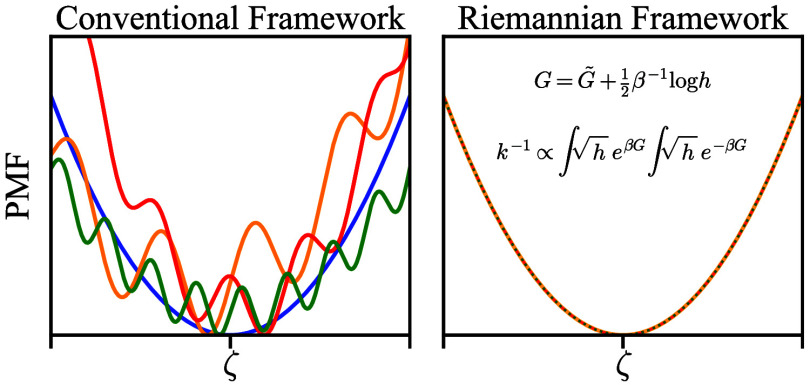

## 1 Introduction

Biomolecular simulations have made enormous progress in recent years. The advent of molecular dynamics (MD) in the study of biomolecular processes^1–3^ has given researchers new molecular level insights into previously unobservable phenomena. MD has overcome^4,5^ the common limitation of experimental techniques which forces the researchers to choose between high-resolution static (such as X-ray crystallography) or low-resolution dynamic (such as single-molecule fluorescence resonance energy transfer spectroscopy) pictures of biomolecular systems. MD does however have a few drawbacks. A key issue is the “time scale gap” in that MD simulations typically have a shorter time scale than many relevant biological phenomena. A related issue is the metastability; the system gets trapped in local free energy minima, preventing the system from evenly visiting the entire free energy landscape. Various enhanced sampling techniques and path-finding algorithms have been developed over the last few decades in order to overcome these limitations.^6–25^ These techniques have proven quite successful for simple toy models (e.g., dialanine peptide^13,23,26–30^ ) and, over the last few decades, have been successfully applied to a wide range of realistic biomolecular processes, including protein folding and binding,^31,32^ conformational transitions,^33–35^ and ligand binding.^36,37^ Despite this significant progress, their application to increasingly complex systems remains a considerable challenge.

Perhaps the most obvious line of attack for solving the sampling problems is simply to have stronger computing power and more efficient or specialized computer hardware. In the past few decades, extraordinary advances have been made in this regard. Current state of the art architectures such as very large^38–41^ or MD specialized^42–44^ supercomputers and GPU-enabled computing^45–49^ have capabilities that allow for simulations with large number of particles to run over long periods of time. The size of supercomputers have also given rise to algorithms developed for speeding up the calculations within brute force MD such as particle mesh Ewald^50^ and dynamic load balancing.^51^ The focus of this article, however, is the enhanced sampling techniques that are specifically based on enhancing the sampling within a statistical mechanical framework, in particular those methods that, in some form or another, use CV within their formalism.

In order to obtain relevant thermodynamic and kinetic^52^ properties of a statistical mechanical system, one must integrate over high dimensional spaces, which requires large sets of independent and identically distributed samples. In order to manage the dimensionality of biomolecular systems, the assumption is often made that the vast majority of the high-dimensional space is practically “empty” (or occupied by microstates of negligible probability) and the occupied space can be approximated by a lower-dimensional manifold which contains all relevant conformations from stable states to important transition states. Ideally, any elementary reaction, can be thought of as a transition between two stable states, characterized by a transition pathway^53,54^ (or committor function^55^).

The dimensionality reduction may be employed explicitly or implicitly in a sampling scheme. For instance, a path-optimization technique^14,29,56–64^ can be thought of as a dimensionality reduction technique, if the optimized transition path can be approximated as a thin transition tube, where the areas outside the tube are associated with low probabilities. In this case, one may parametrize this path and potentially deviation from this path^59,60,65^ and use them as CVs. Other methods attempt to identify the intrinsic manifold by using statistical learning methods such as principal component analysis,^66^ time-lagged independent component analysis,^67^ isomap,^53^ diffusion map,^54,68^ and autoencoders.^69–71^ Often these techniques are used to analyze MD trajectories,^53,54,66,72^ or to build Markov state models^73,74^ or are combined with enhanced sampling as in metadynamics^75,76^ or adaptive biasing force,^77^ or integrated within adaptive sampling frameworks.^78^ In this manuscript, we focus on the geometric structure induced by a chosen set of CVs, rather than on how those CVs are discovered.

Whether a set of CVs is defined in a systematic manner as described above or it is defined intuitively, it is a convenient way of reducing the dimensionality of the configuration space in both free energy calculation methods and path-finding algorithms. Various algorithms have been developed to estimate free energies^6,8,9,13,30,79–84^ or find transition pathways^14,29,56,59–61,85^ in CV spaces. What is mostly missing is a robust theoretical framework that allows for a rigorous treatment of the issues one needs to deal with when working with CVs. For instance, the CVs used in collective-variable-based enhanced sampling methods are often nonlinear transformations of atomic coordinates. This complicates their application as noted previously by Johnson and Hummer,^86^ who show the conventional MFEP obtained from various path-finding algorithms are CV specific and are not invariant under nonlinear coordinate transformations. Such difficulties have often been ignored in the past in the majority of the applications of the collective-variable-based simulations; however, there has been attempts in addressing them as in the aforementioned work^86^ or within the framework of Transition Path Theory.^56,87^ We also previously introduced a Riemannian framework for the rigorous treatment of the collective-variable-based spaces within the context of enhanced sampling and path-finding algorithms,^88^ where the distance, free energy, and MFEP are redefined in a way that are all invariant quantities and do not change under coordinate transformations. Our previous work focused on the thermodynamic characterization of protein dynamics within a Riemannian diffusion model.^88^ We did not previously discuss how to derive rate constants that, like the Riemannian PMF, are invariant under coordinate transformation. Here we extend the formalism in two different directions. First, in Section 2, we show that the Riemannian formulation is more general than its diffusion model manifestation and applies to a broad class of CV-based enhanced sampling and analysis methods. In Section 3, we extend the Riemannian diffusion model by focusing on the kinetics and rate calculation techniques, which we previously did not elaborate on.^88^ In Section 4, we describe how to estimate transition rates in practice, and in Section 5 we use a 2-D toy model to illustrate how the correct geometric treatment of CV spaces affects both thermodynamic and kinetic properties. While the 1-D and 2-D examples clarify the underlying concepts, we also apply the framework to alanine dipeptide in Section 6 to demonstrate its feasibility in a realistic biomolecular system.

## 2 From Euclidean to Riemannian CV Space

Euclidean geometry was developed by the Greek Euclid ca. 300 BC. Early in the 19th century, mathematicians such as Gauss,^89^ Schweikart,^90^ Bolyai^91^ and Lobachevsky^92,93^ began to formulate non-Euclidean geometries. With the work of Bernhard Riemann, specifically his lecture “On the Hypotheses which lie at the Bases of Geometry”,^94^ published first in 1873, geometry started exploring new and more diverse applications. Riemann’s work on generalizing the differential surfaces of R ^3^ led to progress in many fields of science. Further progress upon his ideas allowed for the formulation of Einstein’s General Theory of Relativity^95^ and progress in group theory.^96^

Riemannian geometry provides a robust mathematical framework to develop a formalism for the geometry of CV spaces, that are often defined to reduce the dimensionality of the atomic models of macromolecular systems. For instance, consider a transmembrane protein whose transmembrane helices rotate under certain conditions to allow opening or closing of a gate and transporting materials across the membrane. An intuitive CV for such a system would be the orientation of the transmembrane helices that can be determined using principal axes of the helices or their orientation quaternions.^97,98^ In order to work in these spaces, we must first have a formalism that allows us to answer common questions which are posed in a typical collective-variable-based simulation. Examples of such questions include: what is the distance between two points in the CV space? How is the potential of the mean force (PMF) defined at a given point in the CV space? How does the system diffuses along a transition pathway? How can we estimate the rate of a transition along a transition pathway? The questions have previously been answered within a Euclidean framework but with our Riemannian treatment of the CV space, some of these concepts and quantities need to be revisited. We will begin to answer these questions one at a time.

Imagine a system containing *N* atomic coordinates described by position vector ***x*** under a potential energy surface *V*(***x***). In order to reduce the dimensionality of the system and quantify important functional states, a coarser space is desirable to be defined such that **ξ**: R ^n^ → R ^m^, where *n* = 3*N* (N being the number of particles) and **ξ** is a multidimensional CV. The PMF is typically defined as

1 $$Ã\left(\zeta \right)=-\beta^{-1}\;\log\int \delta \left(\zeta -\xi \left(x\right)\right)\;\exp\left(-\beta V\left(x\right)\right)d^{N}x$$

where *Ã*(**ζ**) is the PMF at any given point **ζ** in the CV space and δ(.) is the conventional Dirac delta function.

The PMF as defined above is sometimes considered as the effective potential energy of the reduced system (i.e, a system which is described by the CV and not the atomic coordinates). For multidimensional CV spaces, the MFEP or other related 1D pathways are often used for extensive sampling and characterization in place of the entire multidimensional space. In other words, the multidimensional CV space can be reduced itself to a 1D curve defined in the CV space.

The above approach is quite common and provides a powerful tool for characterizing the energetics of large biomolecular systems. However, we argue here that the above definitions of PMF and MFEP are not well-suited for the purposes that they are defined for. The main problem with these quantities is that they are not invariant under coordinate transformation. Unlike our previous work,^88^ the discussions in this manuscript do not make any assumption regarding the diffusivity of the effective dynamics in the CV space and are more general. In the next Section, we will discuss the implications of the diffusivity and the Riemannian diffusion model.

Let us consider a simple toy model to clearly illustrate the noninvariance of conventional PMF. Our toy model is a 1D system in thermal equilibrium with a heat reservoir of temperature *T*, where β = (*k*_*B*_ *T*)^−1^ = 1. The system is governed by the potential energy *V*(*x*). The PMF along any CV ξ­(*x*) is defined as

2 $$Ã\left(\zeta \right)=-\log\int_{-\infty }^{\infty }\exp\left(-V\left(x\right)\right)\delta \left(\zeta -\xi \left(x\right)\right)\mathrm{dx}$$

Defining η­(ξ) as the inverse function of ξ­(x) such that *x* = η­(ξ), and assuming d*η*/d*ξ* > 0 everywhere, we can show:

3 Ã(ζ)=−log⁡∫−∞∞exp(−V(η(ξ)))δ(ζ−ξ(η(ξ)))dηdξdξ=−log⁡∫−∞∞exp(−V(η(ξ)))δ(ζ−ξ)dηdξdξ=−log(exp(−V(η(ζ)))dηdξ|ξ=ζ)=V(η(ζ))−log(dηdξ|ξ=ζ)

In other words, if $\eta^{\prime }\left(\zeta \right)=\frac{d\eta }{d\xi }{|}_{\xi =\zeta }$ , we have

4 $$Ã\left(\zeta \right)=V\left(\eta \left(\zeta \right)\right)-\log\left(\eta^{\prime }\left(\zeta \right)\right)$$

Projecting the CV back to the *x* space, we have

5 $$Ã\left(\xi \left(x\right)\right)=V\left(x\right)+\log\left(\xi^{\prime }\left(x\right)\right)$$

where we have used ξ′(*x*) = 1/η′(ζ) since η is the inverse function of ξ.

Relations ([Disp-formula eq4]) and ([Disp-formula eq5]) clearly show that the shape of PMF is not determined by the potential energy of the system only but it also depends on the derivative of the CV. One may use any free energy calculation method (such as umbrella sampling,^6^ metadynamics,^75,76^ or ABF^77^) to calculate the PMF; however, without taking into account the second term in Relation [Disp-formula eq4] or 5, the PMF’s shape or the shape of its projection onto the *x* space, does not represent the underlying potential energy for such a 1D system.

As an example, let us assume *V*(*x*) = *x* ^2^. The shape of PMF may or may not be similar to this potential energy, depending on the definition of CV. Figure 1 shows *V*(*x*) along with the PMF as a function of several CVs (up to an additive constant) that were specifically designed to result in various PMFs. If ξ­(*x*) = *x*, the PMF would be the same as *V*(*x*). However, if ξ­(*x*) = *erf*(*x*), the PMF becomes flat since:

6 $$Ã\left(\zeta \right)=Ã\left(\xi \left(x\right)\right)=V\left(x\right)+\log\left(\xi^{\prime }\left(x\right)\right)=x^{2}+\log\left(\frac{2}{\sqrt{\pi }}\exp\left(-x^{2}\right)\right)=\log\left(\frac{2}{\sqrt{\pi }}\right)$$

Using Relation [Disp-formula eq4], one may solve the following nonlinear differential equation to generate any arbitrary PMF function *Ã*(ζ):

7 $$Ã\left(\zeta \right)=\eta^{2}\left(\zeta \right)-\log\left(\eta^{\prime }\left(\zeta \right)\right)$$

or by some rearrangement:

8 $$\frac{d\eta }{d\zeta }=\exp\left(-Ã\left(\zeta \right)+\eta^{2}\left(\zeta \right)\right)$$

Once the above differential equation is solved for η­(ζ), one can easily find its inverse function ξ­(*x*). We used this procedure using numerical methods to find functions α­(*x*) and β­(*x*) that result in *Ã*(ζ) = −ζ^2^ and *Ã*(ζ) = sin­(10ζ), respectively. Figure 1 illustrates how the PMF, as defined conventionally based on these CVs, could qualitatively behave differently from what we expect intuitively from the underlying energetics (here, the potential energy) of this simple system. This is true when PMF is plotted against ζ (which is generally expected as ζ and *x* are not the same) and more importantly when it is projected back onto the *x* space.

**Figure 1:**
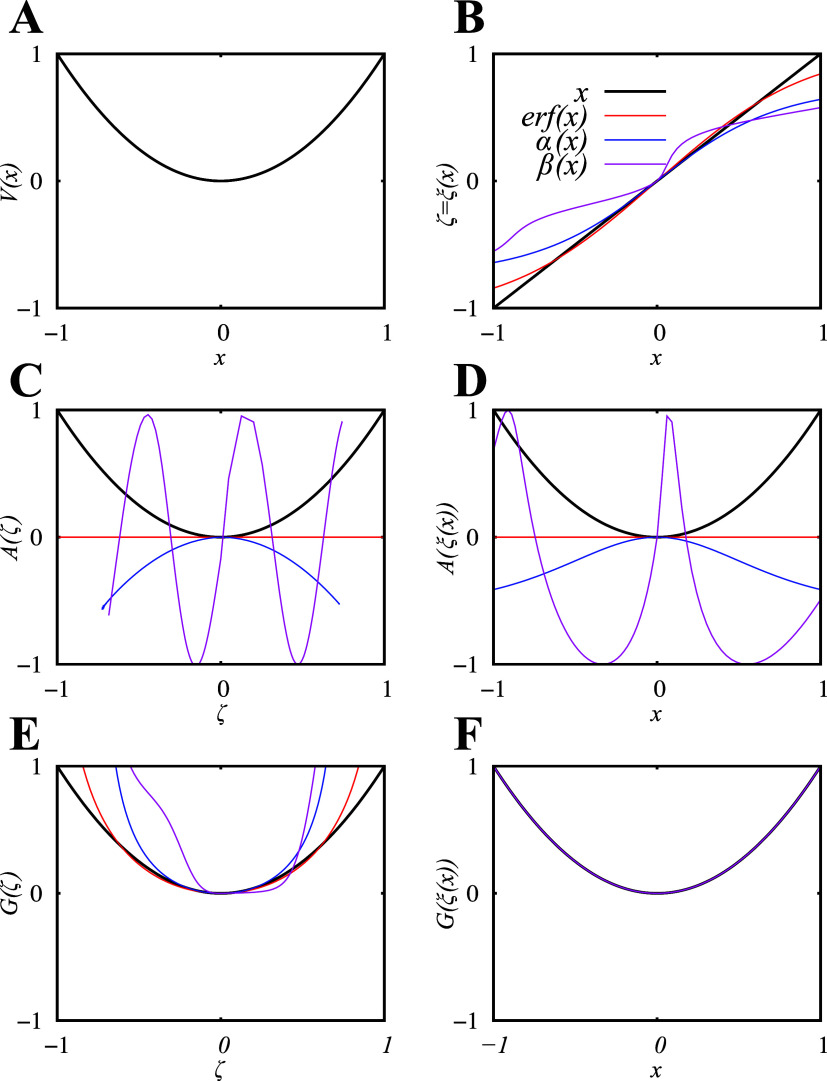
Toy Model: (A) A one-dimensional toy model described by potential energy *V*(*x*) = *x* ^2^. (B) Several CVs (ξ­(*x*)) are used as examples of smooth coordinate transformations ( $x\overset{\xi }{\to }\zeta$ ). (C) The conventional PMF along the CVs defined in (B). (D) The projection of conventional PMF (shown in C) onto the *x* space. (E) The Riemannian PMF along the CVs defined in (B). (F) The projection of Riemannian PMF (shown in E) onto the *x* space (all PMFs are exactly the same when projected onto the *x* space, irrespective of the CV used).

The above example clearly shows that the PMF could be quite misleading if interpreted as a typical potential energy surface, where the minima are considered as locally stable states and the maxima are interpreted as transition states. However, since the CV function ξ­(*x*) is known, log­(ξ′(*x*)) can also be calculated and subtracted from the PMF to result in *V*(*x*). To do this, first one needs to project *Ã*(ζ) onto the *x* space by using ζ = ξ­(*x*) before subtracting log­(ξ′(*x*)) (Relation [Disp-formula eq5]). Alternatively, one may add log­(η′(ζ)) to *Ã*(ζ) to get *V*(η­(ζ)) (Relation [Disp-formula eq4]) and then project that onto *x* to get *V*(*x*). We define an invariant PMF as

9 $$A\left(\zeta \right)=V\left(\eta \left(\zeta \right)\right)=Ã\left(\zeta \right)+\log\left(\eta^{\prime }\left(\zeta \right)\right)$$

Note that *A*(ζ) is a function of ζ similar to conventional PMF but *A*(ξ­(*x*)) = *V*(*x*) for any ξ­(*x*). We argue *A*(ζ) is conceptually more useful than *Ã*(ζ) since its connection to *V*(*x*) is more straightforward and its minima and maxima correctly represent the minima and maxima of *V*(*x*). Figure 1 illustrates how the same CVs used for conventional PMF calculations can also be used to calculate the invariant PMF *A*(ζ). The resulting PMFs all qualitatively look similar but once projected back onto the *x* space, they all result in an identical function, i.e., *V*(*x*).

The above definition of invariant PMF can be reformulated as below

10 $$A\left(\zeta \right)=-\log\int_{-\infty }^{\infty }\exp\left(-V\left(\eta \left(\xi \right)\right)\right)\delta \left(\zeta -\xi \right)d\xi =-\log\int_{-\infty }^{\infty }\exp\left(-V\left(x\right)\right)\delta \left(\zeta -\xi \left(x\right)\right)\xi^{\prime }\left(x\right)\mathrm{dx}$$

Assuming *x* = η­(ζ) is the only solution to ζ = ξ­(*x*) (i.e., assuming ξ is an invertible function of *x*), we can show δ­(ζ – ξ­(*x*)) = δ­(*x* – η­(ζ)) /|ξ′(*x*)| (which is the result of identity δ­(*y*) = ∑_*i*_ δ­(*x* – *x*_*i*_) /|*y*′(*x*_*i*_)|, where *x*_*i*_’s are the roots of *y*(*x*)). Therefore, using Relation [Disp-formula eq10], we have

11 $$A\left(\zeta \right)=-\log\left\langle \delta \left(\zeta -\xi \left(x\right)\right)\xi^{\prime }\left(x\right)\right\rangle =-\log\left\langle \delta \left(x-\eta \left(\zeta \right)\right)\right\rangle$$

After illustrating the noninvariance nature of conventional PMF and showing that the problem can be resolved by modifying the definition of PMF, we can now generalize the definition of PMF to multidimensional spaces. Relation [Disp-formula eq11] is very easily generalizable for a full transformation from n-dimensional ***x*** to m-dimensional **Ξ**(***x***), where we also no longer assume β = 1:

12 $$A\left(Z\right)=-\beta^{-1}\log\left\langle \delta^{\left(m\right)}\left(Z-\Xi \left(x\right)\right)\right|J_{\Xi }\left|\right\rangle =-\beta^{-1}\log\left\langle \delta^{\left(n\right)}\left(x-\eta \left(Z\right)\right)\right\rangle$$

in which *J*_Ξ_ is the determinant of Jacobian ***J***_Ξ_ of transformation **Ξ**, i.e., ${\left[J_{\Xi }\right]}_{\mathrm{ij}}=\frac{\partial \Xi_{i}}{\partial x_{j}}$ where *i* = 1,···, *m* and *j* = 1,···, *n*, ***Z*** is any given point in the CV space, **η**(***Z***) is the inverse map that returns the unique ***x*** coordinates whose CV value is ***Z***. Unfortunately, the full transformation is generally not desirable and **η**(***Z***) is not available.

Of course, *J*_Ξ_, is defined only when *n* = *m*. When a CV **ξ**(***x***) is lower-dimensional than ***x***, one can assume **ξ**(***x***) is part of a full transformation. First, we assume ξ­(***x***) is one-dimensional to simplify the discussion. We can keep the definition of ξ­(***x***) general, yet choose **Ξ**(***x***) to be the full transformation, such that it contains ξ­(***x***) and a (*N* – 1) -dimensional vector orthogonal to ξ­(***x***), denoted by **ϕ**(***x***). Together, **Ξ**(***x***) = (ξ, **ϕ**), and orthogonality implies ***∇*** ξ· ***∇*** ϕ_*i*_ = 0 for all *i*. Here ***∇*** is the gradient in *x*. Now we can write

13 $$A_{\Xi }\left(Z\right)=-\beta^{-1}\;\log\left\langle \delta^{\left(N\right)}\left(Z-\Xi \left(x\right)\right)\right|J_{\Xi }\left|\right\rangle$$

Since the determinant of the product of matrices is equal to the product of their determinants, we can also write

14 $$A_{\Xi }\left(Z\right)=-\beta^{-1}\;\log\left\langle \delta^{\left(N\right)}\left(Z-\Xi \left(x\right)\right)\sqrt{J_{\Xi }^{2}}\right\rangle$$

where $\sqrt{J_{\Xi }^{2}}$ is the determinant of matrix ***J***_Ξ_^2^ (i.e., $\sqrt{J_{\Xi }^{2}}\equiv \sqrt{\det(J_{\Xi }}J_{\Xi }^{T})=\left|J_{\Xi }\right|$ ). Subsequently, one can write

15 $$\exp\left(-\beta A_{\Xi }\left(Z\right)\right)=\left\langle \delta^{\left(N\right)}\left(Z-\Xi \left(x\right)\right)\sqrt{J_{\Xi }^{2}}\right\rangle$$

Since ξ­(***x***) is orthogonal to all ϕ_*i*_(***x***), one can easily show that *J*_Ξ_ = *J*_ξ_ *J*_ϕ_, where *J*_ξ_^2^ = ***∇*** ξ. ***∇*** ξ and *J*_ϕ_^2^ is the determinant of a (*N* – 1) × (*N* – 1) matrix containing elements ***∇*** ϕ_*i*_. ***∇*** ϕ_*j*_. We have

16 $$\exp\left(-\beta A_{\Xi }\left(Z\right)\right)=\left\langle \delta \left(\zeta -\xi \left(x\right)\right)\sqrt{J_{\xi }^{2}}\delta^{\left(N-1\right)}\left(\theta -\phi \left(x\right)\right)\sqrt{J_{\phi }^{2}}\right\rangle$$

where ***Z*** = (ζ, **θ**). The *N*-dimensional invariant PMF can be used to define the 1D invariant PMF *A*(ζ) as

17 $$\exp\left(-\beta A\left(\zeta \right)\right)=\int \exp\left(-\beta A_{\Xi }\left(Z\right)\right)J_{\phi }^{-1}d^{N-1}\phi$$

Note that *J*_ϕ_^–1^ appears in the integral since we have change the variable to ϕ. Inserting into the above relation gives

18 $$\exp\left(-\beta A\left(\zeta \right)\right)=\left\langle \delta \left(\zeta -\xi \left(x\right)\right)\right|J_{\xi }\left|\right\rangle$$

*A*(ζ) is invariant since (1) *A*_Ξ_(***Z***) is invariant and (2) *A*(ζ) does not depend on the choice of **ϕ** as long as **ϕ** is orthogonal to ξ.

Now we define what we refer to as the metric *g*, using the conditional ensemble average of *J*_ξ_:

19 $$g^{-1/2}\left(\zeta \right)=\langle \sqrt{J_{\xi }^{2}}\rangle_{\zeta =\xi \left(x\right)}=\frac{\left\langle \delta \left(\zeta -\xi \left(x\right)\right)\sqrt{J_{\xi }^{2}}\right\rangle }{\left\langle \delta \left(\zeta -\xi \left(x\right)\right)\right\rangle }$$

With the above definition of metric, one can now write

20 $$\exp\left(-\beta A\left(\zeta \right)\right)=\left\langle \delta \left(\zeta -\xi \left(x\right)\right)\right\rangle g^{-1/2}$$

By introducing a fixed reference constant with the same units as *g*, *g*_0_ ≡ *g*(ζ_*ref*_), we can safely take a logarithm:

21 $$A\left(\zeta \right)=-\beta^{-1}\;\log\left\langle \delta \left(\zeta -\xi \left(x\right)\right)\right\rangle +\frac{1}{2}\beta^{-1}\;\log\left(\frac{g\left(\zeta \right)}{g_{0}}\right)\equiv Ã\left(\zeta \right)-\frac{1}{2}\beta^{-1}\;\log\left(g_{0}g^{-1}\left(\zeta \right)\right)$$

The above definition of 1D invariant PMF *A*(ζ) can be generalized to any arbitrary number *d* of dimensions:

22 $$A\left(\zeta \right)=-\beta^{-1}\;\log\left\langle \delta^{\left(d\right)}\left(\zeta -\xi \left(x\right)\right)J_{\xi }\right\rangle \;=-\beta^{-1}\;\log\left(\left\langle \delta^{\left(d\right)}\left(\zeta -\xi \left(x\right)\right)\right\rangle g^{-1/2}\left(\zeta \right)\right)\;=Ã\left(\zeta \right)-\frac{1}{2}\beta^{-1}\;\log\left(g_{0}g^{-1}\left(\zeta \right)\right)$$

The metric inverse *g* ^–1^ is defined to satisfy the relationship *g* ^–1/2^ = ⟨*J*_ξ_⟩_**ζ**=**ξ**(***x***)_. This can be achieved by constructing elements of metric inverse *g* ^–1^ as *g*_*ij*_^–1^ = ⟨(*J*_ξ_^2^)_*ij*_⟩_**ζ**=**ξ**(***x***)_, where matrix *J*_ξ_^2^ is composed of components ***∇*** ξ_*i*_· ***∇*** ξ_*j*_.

The above definition of invariant PMF differs from the conventional definition of PMF as it involves the metric tensor *g*. The metric tensor is a well-known quantity in differential geometry and is needed to do invariant measurements in non-Euclidean spaces. The PMF is only an example of a quantity that can be redefined to be invariant with the help of the metric tensor. The noninvariance of the conventional PMF and any other geometric quantity in the CV space is known, although it is often overlooked. The PMF can be made invariant by adding an additional term, which depends on the derivatives of CVs, as derived above. The difference between the invariant PMF and conventional PMF is related to the Fixman potential as also discussed elsewhere.^99,100^ Note that the Fixman potential was originally developed to relate the PMF associated with a constrained dynamics to that of an unconstrained one.^101,102^ Closely related “geometric/covariant” PMFs have been advocated in recent work.^103–106^ These insert the same metric/Jacobian factor, either to define a Transition State Theory-consistent geometric PMF,^103^ to obtain a CV-form-independent activation free energy via the mass-metric,^104^ or to derive a unique covariant PMF from stochastic dynamics with *g* = *D* ^–1^,^106^ where *D* is the diffusion tensor.

A conceptually more straightforward approach to ensure the invariance of not only the PMF but also any other quantity of interest is to treat the CV space as a Riemannian space. In that view the metric tensor replaces the Euclidean dot product, so all distances, gradients and integrals are performed with *g*. For instance, the ordinary volume element *d* ^*N*^ξ is replaced by the invariant one $d\Omega_{\xi }=\sqrt{g}d^{N}\xi$ ; likewise, the invariant gradient is $\nabla_{\xi }A\left(\zeta \right)=\sum_{j}g^{\mathrm{ij}}\frac{\partial }{\partial \xi_{j}}A\left(\zeta \right){ê}_{i}$ , where ***e* ^**_*i*_’s are unit vectors along ξ_*i*_’s. *g*_*ij*_ = ⟨∂_*k*_ξ_*i*_∂_*k*_ξ_*j*_⟩ measures how much the underlying Cartesian space stretches when mapped into CV space. Riemannian geometry provides the conceptual framework and mathematical tools to treat the nonlinear behavior of curved spaces that are smooth but have potentially different curvatures at different points of space. The rigorous treatment of the CV space fits well within the Riemannian geometry framework. In this framework, the Riemannian PMF is defined exactly the same way as the conventional PMF is defined but the Riemannian Dirac delta function replaces the conventional Dirac delta function:

23 $$A\left(\zeta \right)=-\beta^{-1}\;\log\left\langle \delta_{\zeta }\left(\xi \right)\right\rangle$$

in which δ_**ζ**_(**ξ**) is the Riemannian Dirac delta function. Comparing Relation [Disp-formula eq23] to Relation [Disp-formula eq22], one can make a connection between the Riemannian and conventional Dirac delta functions: δ­(**ζ** – **ξ**(***x***))|*J*_ξ_(***x***)| = δ_**ζ**_(**ξ**(***x***)).

We note that *A*(**ζ**) is the same invariant object (up to notation and a constant) as the “geometric/gauge-invariant” PMF.^99,103,107^ Our approach is not a different correction but a different framework: we treat CV space as a Riemannian manifold, so the profile and the calculus on it (volume element, gradients) are invariant by construction, rather than applying an a-posteriori fix to a Euclidean profile. We also note that the difference between the conventional and Riemannian PMF, $-\frac{1}{2}\beta^{-1}\;\log\left(g_{0}g^{-1}\left(\zeta \right)\right)$ , does not affect free-energy differences obtained from basin integrals when the same CVs cleanly separate the metastable states as discussed elsewhere.^105^ By contrast, pointwise quantities read off a conventional PMF well depths, barrier heights, curvatures-depend on the parametrization of the CV (see Example 1, Figure 1C–D). The practical magnitude of this dependency is determined by the variation of the metric term; for simple CVs such as Euclidean distances, the metric is often nearly constant, resulting in a uniform shift that preserves relative barrier heights. However, for nonlinear reparameterizations or curvilinear CVs, the position-dependent metric can introduce significant corrections. Using the Riemannian PMF (Rels. [Disp-formula eq22]–[Disp-formula eq23]) removes this CV dependency and collapses the profiles across CV choices (See Example 1, Figure 1E–F).

With the same argument made above, one can show the conventional MFEP is not invariant under coordinate transformation as shown by Johnson and Hummer.^86^ Our Riemannian treatment of the CV space, however, provides a robust framework for defining the MFEP in an invariant way.^88^ The MFEP in a Euclidean space is defined as any path whose tangent is parallel to the free energy gradient, i.e., d**ξ**/d*s*∥∇_**ξ**_ *Ã*(**ξ**) with ∇_**ξ**_ *A* = ∑_*i*_ (∂*Ã*/∂ **ξ**) ***e* ~**_*i*_. As *Ã* itself is not invariant, that MFEP is not invariant under coordinate transformation, which questions its importance as a meaningful quantity. The Riemannian framework allows us to define the MFEP simply based on the Riemannian/invariant gradient of Riemannian/invariant PMF. In other words, not only both PMF and its gradient are well-defined, invariant quantities within the Riemannian framework, the Riemannian MFEP that is simply a path parallel to the gradient of PMF, $\frac{d\xi^{i}}{\mathrm{ds}}\propto \sum_{j}g^{\mathrm{ij}}\frac{\partial A}{\partial \xi^{j}}$ , is also well-defined and invariant under coordinate transformation. With the invariant definitions of PMF and MFEP established, we now require an appropriate dynamic model consistent with these definitions. The Riemannian diffusion model, detailed next, addresses precisely this need, providing a robust theoretical basis for kinetic characterization.

## 3 Riemannian Diffusion and Transition Rate Estimation

In the previous Section, we did not make any assumptions regarding the dynamics. However, quantities such as PMF and particularly MFEP are difficult to interpret if the reduced system follows a nondiffusive dynamics in the CV space. The intuitive interpretation of minima and saddle points of PMF representing the stable and transition states and the MFEP representing the most probable pathway relies on the diffusive nature of the effective dynamics. Such a condition is only satisfied if at all with specific choices of CVs. Here, the focus of our discussion is not on how to find such CVs. However, if we can successfully identify a set of CVs such that the projected motion of the system on this reduced CV space is diffusive, we can write

24 $$d\zeta =-\left(\beta D\nabla A\left(\zeta \right)+b\right)dt+\sqrt{2D}dW$$

where *D* is the diffusion constant and *b*_*i*_ = −*Dg* ^*jk*^Γ_*jk*_^*i*^, in which Γ_*jk*_^*i*^’s are Christoffel symbols and Einstein summation convention is used. Note that *D* is not assumed to be position-dependent here. Instead the position dependence is absorbed in metric tensor ***g***. d ***W*** is a Riemannian Wiener process, where ⟨*W* ^*i*^(*t*)⟩ = 0 and ⟨ *Ẇ* ^*i*^(*t*_1_)*Ẇ* ^*j*^(*t*_2_)⟩ = *g* ^*ij*^δ­(*t*_1_ – *t*_2_), where *Ẇ* ^*i*^ is the time derivative of *W* ^*i*^.

Rewriting this equation as Fokker–Planck or Smoluchowski equations,^108^ we have

25 $$\frac{\partial }{\partial t}u\left(\zeta ,t\right)=\beta D\nabla \cdot \left(u\left(\zeta ,t\right)\nabla A\left(\zeta \right)\right)+D\Delta u\left(\zeta ,t\right)$$

which contains the Laplace-Beltrami operator, $\Delta =\nabla \cdot \nabla =\frac{1}{\sqrt{g}}\frac{\partial }{\partial i}\sqrt{g}g^{\mathrm{ij}}\frac{\partial }{\partial j}$ , and *u*(**ζ**, *t*) is the probability of finding the system at **ζ** at time *t* with a boundary condition of *u*(**ζ**, 0) = *u*_0_(**ζ**). This summarizes our Riemannian diffusion model, which was previously introduced in ref^88^ In the following we derive a new relation that allows the estimation of the PMF, metric, diffusion constant, and transition rate from unbiased simulations performed along an approximate MFEP.

We have previously discussed how one can find a Riemannian MFEP using the Riemannian implementation of string method with swarms of trajectories (SMwST).^88^ Let us assume we have found such a pathway, **ξ**(*s*), parametrized by its arclength *s*. At any given point along this path, *ê*_*s*_ is the unit vector parallel to **ξ**(*s*). An *N* – 1-dimensional submanifold (Σ_*s*_) can be defined perpendicular to *ê*_*s*_. Let us assume local coordinates **ζ** = (*s*, **κ**) describes any point on Σ_*s*_. One can write

26 $$\frac{\partial }{\partial t}u\left(\zeta ,t\right)=\beta D\frac{\partial }{\partial s}\left(u\left(\zeta ,t\right)\frac{\partial }{\partial s}A\left(\zeta \right)\right)+D\frac{\partial^{2}}{\partial s^{2}}u\left(\zeta ,t\right)+\beta D\nabla_{\Sigma_{s}}\cdot \left(u\left(\zeta ,t\right)\nabla_{\Sigma_{s}}A\left(\zeta \right)\right)+D\Delta_{\Sigma_{s}}u\left(\zeta ,t\right)$$

where ∇_Σ_*s*__ denotes the ∇operator in the submanifold Σ_*s*_.

Let us now define *U*(*s*, *t*)≡ ∫_Σ_*s*__ *u*(**κ**, *t*) ***dΩ***_**κ**_, where Σ_*s*_ is an arbitrary portion of the subspace of **κ** around its origin. On the MFEP, let us also define a univariate PMF *G*(*s*) = *A*(ξ­(*s*)). Continuing, we can integrate over individual terms in Relation [Disp-formula eq26], where the LHS of the equation becomes $\frac{\partial }{\partial t}U\left(s,t\right)$ and the RHS terms can be approximated as follows. Assuming *u*(**ζ**, *t*) is much larger within a relatively narrow “tube” around the MFEP, we can integrate over the cross section of this tube and the areas around the tube. In other words, we choose Σ_*s*_ to be a portion of the **κ** space that covers the cross section of transition tube that falls within this space as well as some low-probability areas around it. The first term of the RHS of Relation [Disp-formula eq26] will thus be

27 $$\beta D\frac{\partial }{\partial s}\int_{\Sigma_{s}}(u\left(\zeta ,t\right)\frac{\partial }{\partial s}A\left(\zeta \right)d\Omega_{\kappa }=\beta D\frac{\partial }{\partial s}\left(U\left(s,t\right)\frac{\partial }{\partial s}G\left(s\right)\right)$$

where we assume inside a cross section Σ_*s*_ the gradient ∂_*s*_ *A* varies negligibly, so we treat it as a constant equal to its value on the MFEP center line *A*(ξ­(*s*)) = *G*(*s*). The next term becomes $D\frac{\partial^{2}}{\partial s^{2}}U\left(s,t\right)$ after integration. For the third term, we assume the following integral is negligible as ∇_*Σs*_ *A* is nearly zero on the tube wall:

28 $$\beta D\int_{\Sigma_{s}}\nabla_{\Sigma_{s}}\cdot \left(u\nabla_{\Sigma_{s}}A\right)d\Omega_{\kappa }=\beta D\oint_{\partial \Sigma_{s}}u\nabla_{\Sigma_{s}}A\cdot nd\sigma_{\kappa }$$

where the divergence theorem is used and ***n*** is the outward normal in the cross section. Inside the tube and far from its boundary ∇_Σ_*s*__ *A* is replacable with ∇_Σ_*s*__ *G*(*s*) which is zero by definition. Similarly, we can use the divergence theorem to reduce the last term to

29 $$D\int \Delta_{\Sigma_{s}}ud\Omega_{\Sigma_{s}}=D\oint_{\partial \Sigma_{s}}\nabla_{\Sigma_{s}}u\cdot nd\sigma_{\kappa }$$

We assume this term is also negligible since *u* is already vanishingly small at the tube boundary and the normal component of its transverse gradient is negligible. These approximations reduce Relation [Disp-formula eq26] to a one-dimensional diffusion equation in terms of probability density *U*(*s*, *t*) and the univariate PMF (or potential energy) *G*(*s*):

30 $$\frac{\partial }{\partial t}U\left(s,t\right)=\beta D\frac{\partial }{\partial s}\left(U\left(s,t\right)\frac{\partial }{\partial s}G\left(s\right)\right)+D\frac{\partial^{2}}{\partial s^{2}}U\left(s,t\right)$$

The thin transition tube, used to simplify Relation [Disp-formula eq26] is a common assumption made for path-finding algorithms such as string method.^109^ At this point, we have reduced our atomic model, first to a coarse variable space, and then we have focused upon a single dimension, namely the transition pathway of interest.

Relation [Disp-formula eq30] is quite similar to the conventional Smoluchowski equation in a 1D Euclidean space. However, this is due to the fact that we assumed *s* to represent the geodesic distance along the MFEP path. A slightly more general relation can be derived based on ([Disp-formula eq30]) for an arbitrary parameter *r* that merely labels points on the MFEP path. The *r*-space is then endowed with a 1D metric *h*(*r*). We have *ds* ^2^ = *h*(*r*)*dr* ^2^ or $\sqrt{h\left(r\right)}=\frac{\mathrm{ds}}{\mathrm{dr}}$ . The Riemannian Smoluchowsky equation equation can therefore be written in terms of *r* as

31 $$\frac{\partial }{\partial t}U\left(r,t\right)=\beta D\frac{1}{\sqrt{h\left(r\right)}}\frac{\partial }{\partial r}\left(U\left(r,t\right)\frac{1}{\sqrt{h\left(r\right)}}\frac{\partial }{\partial r}G\left(r\right)\right)+D\frac{1}{\sqrt{h\left(r\right)}}\frac{\partial }{\partial r}\left(\frac{1}{\sqrt{h\left(r\right)}}\frac{\partial }{\partial r}U\left(r,t\right)\right)$$

which is slightly different from a conventional 1D position-dependent diffusion equation. Here *D*/*h*(*r*) is equivalent to the conventional 1D position-dependent diffusion constant and *G*(*r*) is the same as the conventional 1D PMF in terms of *r* with an extra term $\frac{1}{2}k_{B}T\log h\left(r\right)$ , which makes the Riemannian PMF independent of the choice of *r*.

Finally, we can be recast the Relation [Disp-formula eq31] as

32 $$\frac{\partial }{\partial t}U\left(r,t\right)=D\frac{1}{\sqrt{h\left(r\right)}}\frac{\partial }{\partial r}\left[e^{-\beta G\left(r\right)}\frac{1}{\sqrt{h\left(r\right)}}\frac{\partial }{\partial r}\left(e^{\beta G\left(r\right)}U\left(r,t\right)\right)\right]$$

Now suppose that one has been able to identify the MFEP without necessarily quantifying the metric. Relation [Disp-formula eq32] provides a framework to determine the dynamics of the system as long as it stays close to the MFEP transion tube. The PMF and metric along *r* (*G*(*r*) and *h*(*r*)) fully describe the diffusive dynamics and the arbitrary diffusion constant *D* simply determines the unit of *h*(*r*). The following section shows how Rel. [Disp-formula eq32] can be discretized and statistically estimated, yielding a practical rate matrix ***R*** that can be used to determine thermodynamic and kinetic quantities such as free energy the *G*(*r*), the metric *h*(*r*), and the mean first passage time (MFPT). However, none of these analyses would be meaningful without first identifying the MFEP.

## 4 Determining the Rate of Transition

Starting from the Riemannian 1D Smoluchowski Rel. [Disp-formula eq32], we adopt Hummer’s Bayesian discretization scheme,^110^ generalized to include the metric *h*(*r*). The Riemannian Smoluchowski Rel. of [Disp-formula eq32] can be discretized such that *r* takes discrete values *r*_*n*_ in the range [*r*_*A*_, *r*_*B*_] on a grid size *N*δ*r* (= *r*_*B*_ – *r*_*A*_) with mesh size δ*r* = *r*_*n*+1_ – *r*_*n*_. Applying a second-order finite difference scheme to the [Disp-formula eq32] gives

33 ∂∂tU(rn,t)=Rn,n+1U(rn+1,t)+Rn,n−1U(rn−1,t)−[Rn+1,n+Rn−1,n]U(rn,t),n=0,...,N−1

where the rate element *R*_*n*, *m*_ is given by

34 $$R_{n,m}=\frac{D}{2\delta r^{2}\sqrt{h_{n}}}\left[\frac{1}{\sqrt{h_{n}}}+\frac{1}{\sqrt{h_{m}}}\right]\exp\left(-\beta \frac{G_{n}-G_{m}}{2}\right)$$

or for the nearest-neighbor by assuming, $\frac{1}{\sqrt{h_{n+1/2}}}\approx \frac{1}{2}\left[\frac{1}{\sqrt{h_{n}}}+\frac{1}{\sqrt{h_{n+1}}}\right]$ , we have

35 $$R_{n,n\pm 1}=\frac{D}{\delta r^{2}\sqrt{h_{n}h_{n\pm 1/2}}}\exp\left(-\beta \frac{G_{n}-G_{n\pm 1}}{2}\right)$$

36 $$R_{n,n\pm 1}=\frac{D}{\delta s_{n}\delta s_{n\pm 1/2}}\exp\left(-\beta \frac{G_{n}-G_{n\pm 1}}{2}\right)$$

By construction, the rate matrix satisfies detailed balance, *R*_*nm*_ *e* ^–*βG*_*m*_^ = *R*_*mn*_ *e* ^–*βG*_*n*_^, and probability conservation, ∑_*m*_ *R*_*nm*_ = 0. The boundary conditions are reflective, with *R*_0,–1_ = *R*_*N*, *N*+1_ = 0. More generally, for any lag time Δ*t* and any time *t*, we have

37 $$U\left(\Delta t+t\right)=e^{R\Delta t}U\left(t\right)$$

In this equation, ***U*** represents a discretized form of transition probabilities, which can be determined empirically. On the other hand, ***R*** is a matrix whose elements are defined by the expression given in Rel. [Disp-formula eq35]. In compliance with the detailed balance condition, ***R*** must be a tridiagonal matrix such that *R*_*n,n*_ = – *R*_*n,n*+1_ – *R*_*n,n*–1_. Now it can be seen that working in a Riemannian CV space is valid for the determination of quantities of interest such as rate, free energy, diffusion, and reaction pathways. However, it should be noted that this kinetic treatment assumes an effective diffusive (Markovian) description in the chosen CVs at an appropriate lag time Δ*t*; if strong memory effects persist at all Δ*t*, a CV diffusion model is not expected to yield quantitatively reliable rates.

The rate matrix **R** can be estimated self-consistently,^110–112^ by maximizing the likelihood of the observed transitions. Let a trajectory snapshot α start in bin *i*_α_ at time *t* and be found in bin *j*_α_ at time *t* + Δ*t*. For a continuous-time Markov process the probability of that single observation equals the corresponding element of the time-propagator **P**(Δ*t*) = exp­(**R**Δ*t*). Assuming the observations are independent, the total likelihood of the data set is the product of those matrix elements: *L* = ∏_α_ [*e* ^***R*** Δ*t*^]_*i*_α_, *j*_α__. Therefore, *G* and *h* can be found both by maximizing the likelihood *L*.

From the optimized **R**, free energy between any two states *n* and *m*, Δ*G*_*nm*_ = *G*_*n*_ – *G*_*m*_ can be calculated from the detailed balance relation:

38 $$\Delta G_{n,m}=-k_{B}T\;\log\left(\frac{R_{n,m}}{R_{m,n}}\right)$$

and the metric at midpoint, $h_{n+1/2}$ , can be estimated as

39 $$h_{n+1/2}=\frac{D^{2}}{\delta r^{4}\sqrt{h_{n}h_{n+1}}R_{n,n+1}R_{n+1,n}}$$

which indicates the metric at the midpoint, $h_{n+1/2}$ , depends on the rate matrix elements and the metric at the adjacent grid points, *h*_*n*_, and *h*_*n*+1_. To obtain a numerical estimate, one may use this relationship within an iterative scheme or assume that *h* is slowly varying such that $h_{n}\approx h_{n+1}\approx h_{n+1/2}$ . Finally, the MFPT for a transition starting in bin *A* and reaching bin *B* along the MFEP, τ_*A*→*B*_, which is the inverse of the rate constant *k*_*A*→*B*_, can be written in closed form as the standard 1D Smoluchowski result:^113–115^

40 $$\tau_{A\to B}\equiv k_{A\to B}^{-1}=\frac{1}{D}\int_{r_{A}}^{r_{B}}d\rho \sqrt{h\left(\rho \right)}e^{\beta G\left(\rho \right)}\int_{r_{A}}^{\rho }d\sigma \sqrt{h\left(\sigma \right)}e^{-\beta G\left(\sigma \right)}$$

Here *r*_*A*_ and *r*_*B*_ denote the positions of the bin A and B along the path coordinate *r*, while symbols ρ and σ are dummy integration variables along this path. The invariant PMF *G*(*r*) is obtained from the rate matrix (Rel. 38) and differs from the conventional PMF by the geometric term $\frac{1}{2}k_{B}T\;\log h\left(r\right)$ . Relation [Disp-formula eq40] is obtained by first writing the Smoluchowski equation for diffusion in the geodesic coordinate *s*, where the invariant potential *G*(*s*) and the standard 1D MFPT expression apply, and then transforming back to the arbitrary path label *r*. This change of variables introduces the metric factors $\sqrt{h\left(r\right)}=\mathrm{ds}/\mathrm{dr}$ in the integrand. The inner integral in Rel. [Disp-formula eq40] accumulates the equilibrium weight of all points between basin A and an intermediate position ρ, i.e., how much probability must flow through ρ, while the outer integral weights each intermediate position by its local resistance to motion. In practice, the double integral can be evaluated with standard quadrature techniques.

## 5 2-D Toy Model Example

To illustrate the practical application of the estimation methods proposed in Section 4 and to compare the conventional and Riemannian frameworks, we examine a 2-D system evolving under an overdamped Langevin equation on the Muller-Brown potential surface.^116^ The potential is defined as

$$V\left(x,y\right)=\alpha \sum_{i=1}^{4}A_{i}\;\exp\left[a_{i}{\left(x-x_{0i}\right)}^{2}+b_{i}\left(x-x_{0i}\right)\left(y-y_{0i}\right)+c_{i}{\left(y-y_{0i}\right)}^{2}\right]$$

with a scaling factor α = 0.1. The parameter sets (*A*_*i*_/kcal mol^–1^, *a*_*i*_, *b*_*i*_, *c*_*i*_, *x*_0*i*_, *y*_0*i*_) are (−200, −1.0, 0.0, −10.0, 1.0, 0.0), (−100, −1.0, 0.0, −10.0, 0.0, 0.5), (−170, – 6.5, 11.0, −6.5, −0.5, 1.5), and (15, 0.7, 0.6, 0.7, −1.0, 1.0) for *i* = 1, 2, 3, and 4, respectively. This potential is shown in Figure 2A.

**Figure 2:**
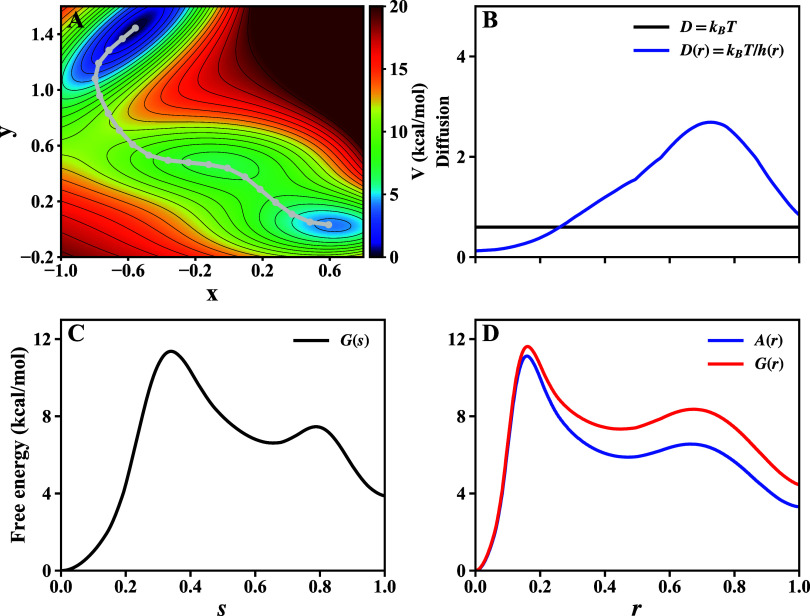
Comparison of thermodynamic and kinetic quantities for a 2-D toy model, analyzed using conventional and Riemannian frameworks. (A) Muller-Brown potential energy surface. The gray line represents the true MFPT connecting the two primary energy minima. (B) Diffusion profiles along the arbitrarily parametrized path *r* in the log-polar (*X*, *Y*) spcae. The black line shows the constant diffusion coefficient *D* used in the Riemannian framework. The blue line shows the position-dependent diffusion coefficient *D*(*r*) = *D*/*h*(*r*). (C) The ground truth free energy profile, *G*(*s*), calculated along the true arc length *s* of the MFEP in the Cartesian (*x*, *y*) space. (D) Free energy profiles calculated along the same arbitrarily parametrized path *r*.

We consider two set of CVs (i) the Cartesian coordinate (*x*, *y*) and (ii) a non-Euclidean log-polar space (*X*, *Y*) where $X=\ln\left(\sqrt{x^{2}+y^{2}}\right)$ and *Y* = arctan­(*y*, *x*). To construct a 1D model of the system’s thermodynamics and kinetics, the first step is to identify the MFEP. The MFEP in the Cartesian coordinate space, (*x*, *y*) was previously identified for this toy model using the SMwST, yielding a coarse-grained pathway of 20 centers^88^ shown in Figure 2A. The MFEP in the (*X*, *Y*) can be identify with Reimannian version of SMwST as discussed in our previous work,^88^ but here our primary goal is to demonstrate that for a given path, even one parametrized by an arbitrary or geometrically incorrect coordinate, the Riemannian analysis framework proposed in Section 4 can recover the thermodynamics and kinetics property the same as conventional one.

To construct a robust 1D model suitable for rate analysis, a higher-resolution path is necessary to satisfy the Markovian assumption. We prepared two distinct high-resolution paths, each discretized into 1000 bins. The first path serves as our “ground truth” and was generated by reparameterizing the initial MFEP by its true Euclidean arc length, *s*, in the (*x*, *y*) space. The second path, designed to test the analysis frameworks, was generated by first transforming the (*x*, *y*) coordinates into (*X*, *Y*), then reparameterized using an intentionally incorrect Euclidean metric in this space, defining an arbitrary path coordinate denoted as *r*, such that *dr* ^2^ = *dX* ^2^+*dY* ^2^. We not that the correct distance element in the (*X*, *Y*) is $\mathrm{ds}=\exp\left(X\right)\sqrt{{dX}^{2}+{dY}^{2}}$ . Using these paths, we constructed ideal rate matrices for three distinct cases: (1) Using the ground truth path parametrized by the true arc length, *s*, in the (*x*, *y*) space, we built a rate matrix using the true potential, *G*(*s*) = *V*(*x*, *y*) and a constant diffusion *D* = *k*_B_ *T*, δ*s* = 1/1000 using

$$R_{n,m}=\frac{D_{n}+D_{m}}{2\delta s^{2}}\left[-\frac{\beta }{2}\left(G_{n}-G_{m}\right)\right]$$

This case serves as our reference for the correct physical properties; (2) Using the arbitrarily parametrized path *r* in the (*X*, *Y*) space, we built a rate matrix based on the conventional framwork using *A*(*r*) = *V*(*X*, *Y*) – 2*k*_B_ *T X* and a position-dependent diffusion coefficient, *D*(*r*) = *D*/*h*(*r*), where *D* = *k*_B_ *T* and *h*(*r*) = exp­(2*X*), and δ*r* = 1/1000. (3) Finally, using the same arbitrarily parametrized path *r* from case 2, we built an ideal rate matrix. As this matrix describes the same underlying physics as in case 2, it is constructed to be identical. We then applied the estimation procedure from Section 4 to this rate matrix to determine the Riemannian PMF, *G*(*r*), and the 1D metric, *h*(*r*), namely via Rel. [Disp-formula eq38] and 39. From the resulting rate matrices, we estimated the free energy and MFPT to compare the outcomes of the three frameworks.

Figure 2C,D show the calculated free energy profiles. Our analysis reveals that first, for a given, arbitrarily parametrized path in the non-Euclidean (*X*, *Y*) space, both the conventional and Riemannian analysis frameworks yield identical *G*(*r*) and MFPT of 1.9 × 10^6^ (time unit). These results confirm the mathematical equivalence of the two formalisms: the geometric information encoded in the Riemannian 1D metric *h*(*r*) is implicitly captured by the position-dependent diffusion *D*(*r*) in the conventional treatment. However, calculated MFPT is still different from the ground truth value of 1.3 × 10^6^ (time unit) obtained from the true MFEP in the Cartesian space. This discrepancy arises because the path analyzed in the (*X*, *Y*) space is not the true MFEP, but rather a nonoptimal pathway that results from incorrectly treating the curved space as Euclidean. This demonstrates the central advantage of the Riemannian framework: while both analysis methods are self-consistent, only a Riemannian approach to path-finding can guarantee the identification of the correct, physically meaningful transition path, thereby leading to accurate predictions of kinetic properties like the MFPT.

## 6 Biomolecular Example: Alanine Dipeptide

While the toy model above highlights the advantages of the Riemannian framework in a controlled setting, we now demonstrate its use for a biomolecular system in which projected dynamics in CV space may not be strictly Markovian. As an illustrative example, we consider alanine dipeptide (*N*-acetyl-*N′*-methylalaninamide, NANMA) in vacuum, which exhibits well-characterized metastability in the (ϕ,ψ) dihedral space (Figure 3A). We focus on the rare transition between the *C*_7_^*eq*^ and *C*_7_^*ax*^ basins.

**Figure 3:**
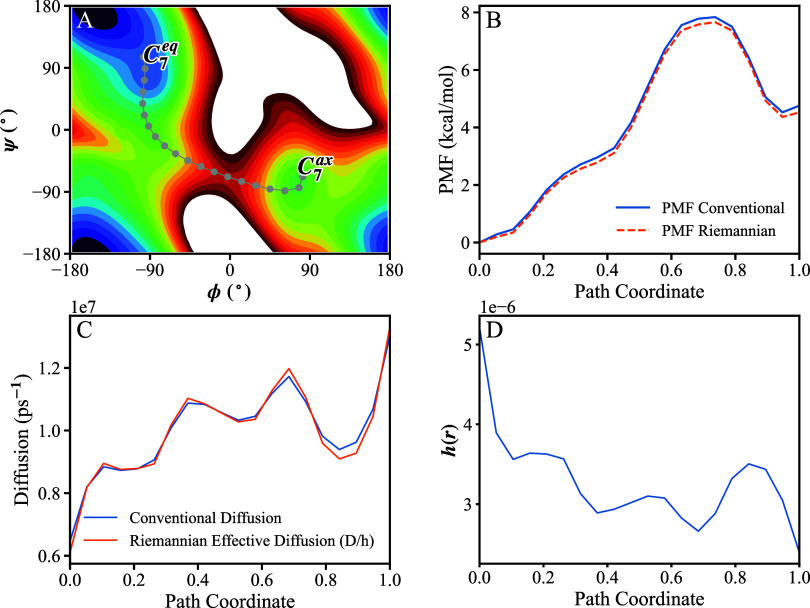
Biomolecular example (alanine dipeptide): Comparison of thermodynamic and kinetic quantities for the C_7_^*eq*^ → C_7_^*ax*^ transition, analyzed using conventional and Riemannian frameworks. (A) Two-dimensional free-energy surface in the (ϕ, ψ) space; the gray curve shows a transition pathway estimated as MFEP, discretized into 20 equally spaced bins. (B) PMF profiles along the path coordinate (*r* = 0 and *r* = 1 correspond to C_7_^*eq*^ and C_7_^*ax*^, respectively), computed using the conventional definition and the Riemannian definition. (C) Diffusion profiles inferred along the same discretized pathway: the conventional position-dependent diffusion *D*(*r*) and the Riemannian effective diffusion *D*/*h*(*r*). (D) The corresponding one-dimensional metric *h*(*r*) estimated along the pathway.

A pathway connecting these basins was first identified using the string method with swarms of trajectories.^117^ Treating this pathway as an approximation to the MFEP in the chosen CV space, we discretized it into a set of centers and estimated a lag-time dependent rate matrix, *R*_Δ*t*_, from short unbiased MD trajectories initiated from these centers (see Supporting Information (SI) for simulation details). Because memory effects can be present after projection to CV space, the resulting kinetic model generally depends on the chosen lag time. Here, to illustrate the method and enable a controlled comparison between the conventional and Riemannian analyses, we select Δ*t* = 10 fs, for which we hypothesize that the relevant slow modes have stabilized, consistent with an effective Markovian description on the discretized path. In general, the reliability of such a kinetic model can be assessed by monitoring the stability of implied time scales or metric over a range of lag times; for the present system, results for different lag time provided in Figure S1.

The free-energy profiles obtained from the conventional definition and from the invariant Riemannian definition are shown in Figure 3B and are nearly identical. This indicates that, for this particular transition pathway, the impact of the Riemannian framework on the PMF is small relative to the barrier height (or is effectively absorbed when the path is well optimized); therefore, the conventional and Riemannian PMFs need not differ substantially in all practical cases. The distinction emerges more clearly in the dynamical interpretation. In the conventional framework, variations in the estimated transition rates along the path are commonly interpreted as a position-dependent diffusion coefficient, *D*(*r*) (Figure 3C). In the Riemannian framework, the same variations are instead attributed to the geometric metric *h*(*r*), which quantifies local stretching (or resistance) along the path. Consistently, plotting the effective diffusion predicted by the Riemannian model, *D*(r)∝1/*h*(*r*), reproduces the shape of the conventional diffusion profile.

Finally, we computed the MFPT for the *C*_7_^*eq*^ → *C*_7_^*ax*^ transition. The conventional and Riemannian analyses yield comparable time scales (approximately 110 and 90 ns, respectively), consistent with their mathematical equivalence for a fixed discretized pathway. Importantly, the Riemannian decomposition provides a clearer physical interpretation by separating geometric effects (encoded in *h*) from energetics (encoded in *G*): what appears as variable diffusion in the conventional treatment can be understood as a consequence of curvature and stretching in CV space along the transition path.

## 7 Summary and Conclusions

In this study, we presented a generalized Riemannian framework that rigorously addresses the noninvariance issues inherent to conventional definitions of thermodynamic and kinetic properties in CV spaces. By using Riemannian geometry, we introduced robust definitions of PMF, MFEP, and diffusion models that remain invariant under nonlinear coordinate transformations. Through detailed theoretical derivations, we demonstrated how conventional Euclidean assumptions can introduce coordinate-dependent artifacts, highlighting the necessity of a proper geometric treatment.

The central thermodynamic result is the relationship between the conventional PMF *Ã*(ζ) and the invariant (Riemannian) PMF *A*(ζ), which differ by an explicit metric correction term (eqs 22 and 23). This expression provides a direct estimate of the potential magnitude of coordinate-induced distortions: when the metric varies weakly over the relevant region of CV space, conventional PMFs are often practically reliable up to an (approximately) constant offset, whereas stronger metric variations can lead to non-negligible changes in the apparent profile and barrier heights. On the kinetic side, we formulated the effective diffusive description in CV space in a coordinate-consistent manner (eqs 24 and 25). Building on this, we presented a practical path-based strategy in which the dynamics are discretized along a pathway and represented by a nearest-neighbor rate model, from which free-energy differences and MFPTs can be computed (eqs 35, 36, 37, 38, 39, and 40).

Using 1D and 2-D representative examples, we explicitly illustrated the practical consequences of neglecting CV space geometry, revealing that a correct Riemannian approach accurately reproduces invariant free energy landscapes and consistent kinetic predictions, whereas conventional methods may yield biased estimates, depending on the choice (and parametrization) of the CVs. This framework thus not only resolves fundamental theoretical inconsistencies but also significantly enhances the interpretability and reliability of biomolecular simulations. Future work will explore the application of this Riemannian formalism to more complex biomolecular systems to further validate its broad applicability.

## Supplementary Material



## Data Availability

The data that support the findings of this study are available from the corresponding author upon request

## Notes

Simulation details for the alanine dipeptide example, along with the rate matrix estimation procedure and the computation of the PMF, diffusion, and metric. The SI also includes a figure comparing the PMF, diffusion, and metric across different lag times in the conventional and Riemannian frameworks (PDF)
