# Supplementary material for: Thermodynamic and Kinetic Analysis of Molecular Conformational Dynamics in a Riemannian Framework
Source: J Phys Chem A. 2026 Jan 26;130(5):1220–32. doi: 10.1021/acs.jpca.5c05362 (PMC12884531; doi:10.1021/acs.jpca.5c05362)
Supplement: Supplementary file 1 [file jp5c05362_si_001.pdf]

Thermodynamic and Kinetic Analysis of Molecular Conformational Dynamics in a Riemannian Framework: Supporting Information

Ashkan Fakharzadeh,[†,‡] Curtis Goolsby,[¶] Emad Tajkhorshid,[†,‡,§] and Mahmoud Moradi[*,¶]

†*Theoretical and Computational Biophysics Group, NIH Resource for Macromolecular Modeling and Visualization, Beckman Institute for Advanced Science and Technology, University of Illinois Urbana-Champaign, Urbana, IL 61801, USA*

‡ *Department of Biochemistry, and Center for Biophysics and Quantitative Biology, University of Illinois Urbana-Champaign, Urbana, IL 61801, USA*

¶ *Department of Chemistry and Biochemistry, University of Arkansas, Fayetteville, AR 72701, USA*

§*Department of Chemistry, University of Illinois Urbana-Champaign, Urbana, IL 61801, USA*

E-mail: moradi@uark.edu

Alanine Dipeptide Simulations

Alanine dipeptide simulations in vacuum were carried out using NAMD 3.0.[1] The simulation setup followed the protocol described in Ref.[2] The system was modeled using the CHARMM22 force field. The temperature was maintained at 300 K using a Langevin thermostat with a damping coefficient of 10 ps^{-1}. A time step of 0.5 fs was used to integrate the equations of motion.

The two-dimensional free energy landscape in terms of the dihedral angles (ϕ, ψ), shown in the main manuscript, was obtained from a 2 μs On-the-fly Probability Enhanced Sampling (OPES) simulation,[3,4] using a barrier parameter of 20 kcal/mol. The minimum free energy path (MFEP) was estimated using the String Method with Swarms of Trajectories (SMwST),[5] as detailed in Ref.[2] First, an initial string of 20 images in the (ϕ, ψ) Collective Variable (CV) space was generated using a post-hoc string method[6] applied to configurations sampled from the OPES simulation. This initial string served as the starting point for the SMwST optimization.[5] The final path and the positions of the 20 images were obtained after 500 iterations, using 20 trajectory copies per image. Each swarm trajectory consisted of 20 MD steps, preceded by 2,000 steps of restrained equilibration with a force constant of 0.5 kcal mol^{-1} deg^{-2}. The final string centers were computed by averaging the string positions over the last 100 iterations. This optimized string was then reparameterized to 20 equally spaced centers defining the transition tube.

Rate Matrix Estimation

To estimate the rate matrices, we first discretized the trajectories onto the transition path. Trajectories were assigned to the nearest bin center (Voronoi tessellation) using the PyEMMA software.[7] To strictly enforce the validity of the 1D approximation, we pruned the data by discarding any trajectory segments that deviated farther than 50° (~ 0.87 rad) from the nearest string center, effectively restricting sampling to a defined transition tube.

Count matrices $C(\Delta t)$ were generated by counting transitions between bins at various lag times Δt. From these count matrices, rate matrices R were estimated using a diagonal adjustment algorithm.[8]

PMF, Metric, and MFPT Calculation

Using the optimized rate matrices, the invariant Riemannian PMF $G(r)$ and the metric $h(r)$ were calculated using Rels. 38–39. The Mean First Passage Time (MFPT) was estimated by numerically evaluating the double integral (Rel. 40).

For the purpose of illustration in Fig. 3, the reference constant D in the Riemannian framework was fixed by a global normalization so that the effective Riemannian diffusion $D/h(r)$ matches the overall scale of the conventional position-dependent diffusion profile inferred from the same rate matrix.

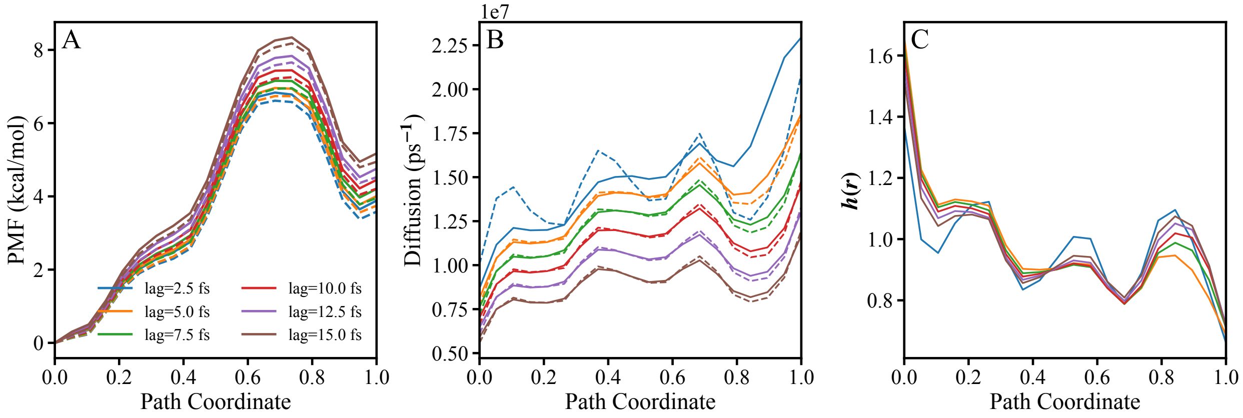

Figure S1: Lag-time dependence along the alanine dipeptide transition path. (A) PMF; (B) 1-D diffusion profile $D(r)$; (C) 1-D Metric $h(r)$ along the path coordinate. Solid and dashed curves correspond to the conventional and Riemannian analyses, respectively, and colors indicate the lag time Δt used to estimate the rate matrix.

References

[S1] Phillips, J. C. et al. *The Journal of Chemical Physics* **2020**, *153*, 044130.

[S2] Tang, C.; Chen, H.; Tajkhorshid, E.; Roux, B.; Chipot, C. *The Journal of Physical Chemistry B* **2025**,

[S3] Invernizzi, M.; Parrinello, M. *Journal of Physical Chemistry Letters* **2020**, *11*, 2731–2736.

[S4] Invernizzi, M.; Parrinello, M. *Journal of Chemical Theory and Computation* **2022**, *18*, 3988–3996.

[S5] Pan, J.; Tristram-Nagle, S.; Kucerka, N.; Nagle, J. F. *Biophysical Journal* **2008**, *94*, 117–124.

[S6] Moradi, M.; Enkavi, G.; Tajkhorshid, E. *Nature Communications* **2015**, *6*.

[S7] Scherer, M. K.; Trendelkamp-Schroer, B.; Paul, F.; PÃl'rez-HernÃąndez, G.; Hoffmann, M.; Plattner, N.; Wehmeyer, C.; Prinz, J.-H.; NoÃl', F. *Journal of Chemical Theory and Computation* **2015**, *11*, 5525–5542.

[S8] Goolsby, C.; Losey, J.; Fakharzadeh, A.; Xu, Y.; Düker, M.-C.; Getmansky Sherman, M.; Matteson, D. S.; Moradi, M. *Journal of Physical Chemistry A* **2023**, *127*, 5745–5759.
